# Evaluation of two formulations of adjuvanted RTS, S malaria vaccine in children aged 3 to 5 years living in a malaria-endemic region of Mozambique: a Phase I/IIb randomized double-blind bridging trial

**DOI:** 10.1186/1745-6215-8-11

**Published:** 2007-03-26

**Authors:** Eusebio V Macete, Jahit Sacarlal, John J Aponte, Amanda Leach, Margarita M Navia, Jessica Milman, Caterina Guinovart, Inacio Mandomando, Yolanda López-Púa, Marc Lievens, Alex Owusu-Ofori, Marie-Claude Dubois, Conor P Cahill, Marguerite Koutsoukos, Marla Sillman, Ricardo Thompson, Filip Dubovsky, W Ripley Ballou, Joe Cohen, Pedro L Alonso

**Affiliations:** 1 Centro de Investigação em Saúde de Manhiça (CISM), Manhiça, Mozambique; 2 Centro de Salud Internacional, Hospital Clínic/Institut d'Investigacions Biomèdiques August Pi i Sunyer (IDIBAPS), Universitat de Barcelona, Barcelona, Spain; 3 Direcção Nacional de Saúde, Ministerio de Saúde, Maputo, Mozambique; 4 Faculdade de Medicina, Universidade Eduardo Mondlane, Maputo, Mozambique; 5 Instituto Nacional de Saúde, Ministerio de Saúde, Maputo, Mozambique; 6 GlaxoSmithKline Biologicals, Rixensart, Belgium; 7 PATH Malaria Vaccine Initiative (MVI), Bethesda, MD, USA

## Abstract

**Background:**

Previous trials of the RTS, S malaria candidate vaccine have shown that this vaccine is safe, tolerated and immunogenic. The development plan for this vaccine aims at administering it in the first year of life through the Expanded Program on Immunization (EPI). The objective was to evaluate the safety and reactogenicity of RTS, S/AS02D (0.5 ml dose), a pediatric formulation of GlaxoSmithKline Biologicals' current malaria candidate vaccine RTS, S/AS02A (0.25 ml dose). A 0.5 ml dose of AS02D is composed of the same active ingredients in the same quantities as in a 0.25 ml dose of AS02A and has been developed to be easily introduced into routine EPI practices.

**Methods:**

We performed a phase I/IIb randomized double-blind bridging study in a malaria-endemic region of Mozambique, to compare the safety and immunogenicity of both candidate vaccines with the aim of replacing RTS, S/AS02A with RTS, S/AS02D as the candidate pediatric vaccine. 200 Mozambican children aged 3 to 5 years were randomized 1:1 to receive one of the 2 vaccines according to a 0, 1, 2 month schedule.

**Results:**

Both vaccines were safe and had similar reactogenicity profiles. All subjects with paired pre and post-vaccination samples showed a vaccine response with respect to anti-circumsporozoite (CS) antibodies irrespective of initial anti-CS serostatus. Geometric mean titers (GMTs) were 191 EU/ml (95% CI 150–242) in recipients of RTS, S/AS02D compared to 180 EU/ml (95% CI 146–221) in recipients of RTS, S/AS02A. For the anti-hepatitis B surface antigen (HBsAg), all subjects were seroprotected at day 90, and the GMTs were 23978 mIU/ml (95% CI 17896–32127) in RTS, S/AS02D recipients and 17410 mIU/ml (95% CI 13322–22752) in RTS, S/AS02A recipients. There was a decrease in anti-CS GMTs between months 3 and 14 in both groups (191 vs 22 EU/mL in RTS, S/AS02D group and 180 vs 29 EU/mL in RTS, S/AS02A group).

**Conclusion:**

Our data show that the RTS, S/AS02D is safe, well tolerated, and demonstrates non-inferiority (defined as upper limit of the 95% confidence interval of the anti-CS GMT ratio of RTS, S/AS02A to RTS, S/AS02D below 3.0) of the antibody responses to circumsporozoite and HBsAg induced by the RTS, S/AS02D as compared to the RTS, S/AS02A.

## Background

Malaria is still leading the list of killer diseases in Africa. Two million children die annually from this disease [1,2] primarily due to infection with *Plasmodium falciparum*. Currently, malaria control strategies are chiefly based on the early diagnosis and treatment of infected individuals. While preventive measures such as vector control and insecticide treated bednets remain indispensable, a vaccine is viewed as an essential part of the long-term strategy to control malaria, especially in Africa, where ninety per cent of all deaths due to malaria occur.

More than 50 candidate vaccines are currently under development [3,4]. GlaxoSmithKline (GSK) Biologicals has developed in collaboration with the Walter Reed Army Institute of Research an adjuvanted candidate malaria vaccine, based on the RTS, S antigen. Since 2001, trials of this vaccine in children have been conducted under a partnership agreement between GSK and the PATH Malaria Vaccine Initiative (MVI). The goal of this partnership is to develop the vaccine for the routine immunization of infants and children living in malaria endemic regions of Africa.

The RTS, S candidate vaccine consists of sequences of the circumsporozoite (CS) protein and the hepatitis B surface antigen (HBsAg) (RTS, S) formulated with AS02 (proprietary oil in water emulsion, MPL^® ^and QS21 immunostimulants). Adjuvanted vaccines based on RTS, S would potentially offer protection against malaria disease due *to P. falciparum*. In addition, studies with the RTS, S/AS02A candidate in African children have shown that the vaccine stimulates the production of high anti-HBsAg antibody titers [5-7].

Previous trials have shown that this vaccine is safe, well tolerated, and immunogenic in malaria-naive [8], semi-immune adults [9], in children aged 6 to 11 years and 1 to 5 years in the Gambia [5]. Safety and proof-of-concept of efficacy were demonstrated in clinical trials conducted in children aged 1 to 4 years in Mozambique [6,7].

Trials of RTS, S candidate vaccine in children under the age of 6 years have most commonly used a fractional dose of the 0.5 ml adult formulation of the RTS, S/AS02A vaccine. An aim of the development plan for this candidate vaccine is to administer it in the first year of life with other vaccines given as part of the Expanded Program on Immunization (EPI) of the World Health Organization (WHO). Currently most injected vaccines given as part of the EPI schedule are administered at a dose volume of 0.5 ml. This study compared the previously tested RTS, S/AS02A vaccine with a new formulation RTS, S/AS02D. A 0.5 ml dose of RTS, S/AS02D is composed of the same active constituents as in a 0.25 ml dose of RTS, S/AS02A, but the final volume has been adjusted to be compatible with the auto-disable syringes used in the EPI program. This trial serves as a clinical bridge of the new EPI-compatible formulation RTS, S/AS02D to the formulation that has demonstrated efficacy in Mozambican children RTS, S/AS02A.

## Methods

### Study area

The study was conducted at the Centro de Investigação em Saúde de Manhiça (Manhiça Health Research Centre – CISM), in the Manhiça district (capital Manhiça, 25°24' S, 32°48' E, 50.5 m above sea level), in southern Mozambique. Manhiça lies 80 km north of the Mozambican capital Maputo. The area is a flat savannah with two distinct seasons: a warm season between November and April, when most of the rains occur, and a cool and generally dry season during the rest of the year. The average annual rainfall is 1100 mm. Moderately intense malaria transmission, mainly caused by *P. falciparum*, is perennial and marked by substantial seasonality [10]. The estimated average Entomological inoculation rate (EIR) is 38 infective bites per person per year, and *Anopheles funestus *is the main vector [6].

The CISM is the first peripheral research centre of the Mozambican Ministry of Health and is adjacent to the Manhiça Hospital. A continuous Demographic Surveillance System has been set up since mid-1996 in an area of approximately 10 km around the centre, referred to as the study area, where participants to this study were recruited. Currently the study area covers 500 km2 and 70,000 inhabitants.

The Manhiça Hospital is a referral health facility for the Manhiça district. It has a 110-bed inpatient ward, an outpatient department, a maternal and child health clinic and an emergency room. Malaria, acute respiratory infection, and malnutrition remain the most important causes of illness and death in children < 5 years old. Currently it also carries out preventive and treatment programs for HIV/AIDS. The district health network consists of a further eight peripheral health posts and a rural hospital. Additional information on the area can be found elsewhere [6,11].

### Study population

In this phase I/IIb study, 200 healthy children aged 3 to 5 years were recruited into two equal groups at a single centre in Mozambique. Non-coercive methods were used during the recruitment. The protocol was approved by the national Mozambican ethics review committee; the Hospital Clinic of Barcelona ethics review committee, and the Human Subjects Protection Committee of PATH. The trial was undertaken according to the International Conference on Harmonization Good Clinical Practice guidelines and was monitored by GSK Biologicals. A Local Safety Monitor and a formally constituted Data Safety Monitoring Board (DSMB) operating under a charter, closely reviewed the conduct and results of the trial.

The selection of the potential candidates was based on the demographic surveillance system in place in the study area, covering a population of around 70 000 people. Lists of potentially eligible resident children were produced from this census. Prior to the enrolment, information sheets and invitations to participate in the trial were delivered and read to parents and guardians of these children. The information sheets were in Portuguese or the local language (Shangana or Ronga). Criteria for recruitment included confirmed residency in the study area and full immunisation with EPI vaccines as indicated on the child's health card. Only children whose parents or guardians had signed or thumb-printed a consent document were screened for eligibility into the study. Standard inclusion and exclusion criteria were checked. At screening, a brief medical history and a medical examination of each child were performed. Blood samples were taken by finger prick for hematology and biochemistry tests. On the day of first vaccination, children were randomized in a 1:1 fashion to receive three doses of either RTS, S/AS02A candidate malaria vaccine or RTS, S/AS02D (GlaxoSmithKline Biologicals, Rixensart, Belgium) according to a 0, 1, 2 months vaccination schedule.

**Figure 1:**
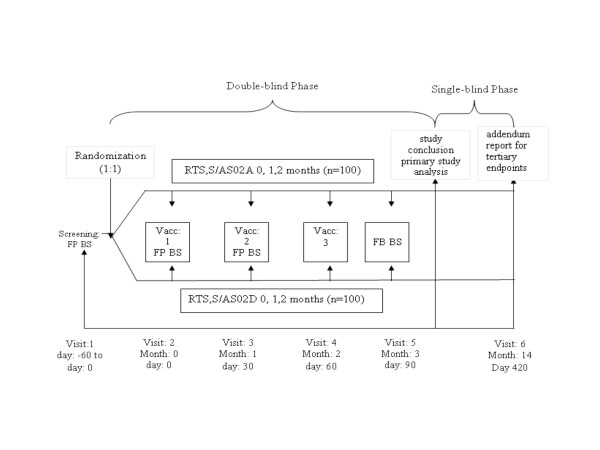
**Overall Study Design**. Key: Vacc: Vaccination; FP: Fingerprick; BS: Bloodsample.

The candidate vaccine was administered in a double-blind manner (observer blinded, participant blinded). Details about study design are presented in Figure 1.

### Vaccines

#### The RTS, S/AS02A vaccine

The RTS, S antigen was presented as lyophilized antigen pellet containing 62.5 μg of RTS, S with 12.6 mg of sucrose as cryoprotectant per 3 mL monodose vial. The pellet was reconstituted with adjuvant in liquid form and 0.5 mL of reconstituted vaccine contains 50 μg RTS, S.

The adjuvant AS02A contains 50 μg MPL^®^, 50 μg QS-21 (QS21 is a triterpene glycoside purified from the bark of *Quillaja saponaria*) and 250 μl of a proprietary oil-in-water-emulsion in phosphate buffered saline per 0.5 mL, presented in prefilled syringes (PFS). In this study a final dose volume of 0.25 mL of the vaccine, was administered.

#### RTS, S/AS02D vaccine

The RTS, S antigen was presented as lyophilized antigen pellet contains 31.25 μg of RTS, S with 12.6 mg of sucrose as cryoprotectant per 3 mL monodose vial. The pellet was reconstituted with adjuvant in liquid form and 0.5 mL of reconstituted vaccine contains 25 μg RTS, S. The AS02D contains 25 μg of MPL^®^, 25 μg QS21 (QS21 is a triterpene glycoside purified from the bark of *Quillaja saponaria*) and 125 μl of a proprietary oil-in-water emulsion in phosphate buffered saline per 0.5 mL, presented in prefilled syringes. A dose of 0.5 mL was delivered.

The RTS, S/AS02A (0.25 mL dose) was supplied such that the reconstituted vaccine provided a 0.5 mL volume. One 0.25 mL dose was aspirated from each vial and used. The RTS, S/AS02D (0.5 mL dose) was supplied such that the reconstituted vaccine volume provided a 0.5 mL pediatric dose. One 0.5 mL dose was aspirated from each vial and used. Both vaccines had the same aspect, which was an opaque milky liquid.

### Assessment of safety and reactogenicity

Safety was assessed by the measurement of hematological and biochemical parameters and the recording of solicited local and general symptoms during a 7-day follow-up period after each vaccination, and of unsolicited non-serious adverse events (AE) in the 30 days post-vaccination, as well as any serious adverse event (SAE) occurring throughout the study.

Blood samples were taken by finger prick at screening one month after the first and last doses and at the end of study follow-up (month 14), for the assessment of safety parameters (complete blood count, hematocrit, creatinine, ALT and bilirubin), and also for testing for HBsAg at screening. HBsAg testing was carried out at the Microbiology Service of the Hospital Clínic in Barcelona, using a test kit (ETI-MAK-4 DIASORIN or equivalent) according to the manufacturer's instructions and laboratory SOPs.

Local symptoms included pain or swelling at the injection site, and general symptoms included fever (defined as axillary temperature ≥ 37.5°C, taken by the field worker), drowsiness, loss of appetite and irritability or fussiness. Grade 3 fever was defined as axillary temperature > 39.0°C. Grade 3 pain was defined as crying when the limb was moved or spontaneously painful limb; grade 3 fussiness as crying that could not be comforted or fussiness that prevented normal activity; grade 3 loss of appetite as not eating at all, and for all other symptoms, grade 3 intensity was defined as preventing normal everyday activities.

Trained field workers, under the supervision of the Principal Investigator, visited each enrolled subject at daily intervals for days 1 to 6 after each vaccination. If the field worker found any grade 3 solicited general or unsolicited symptoms, the volunteer was brought to the Manhiça Hospital for examination by the Principal Investigator or other designated staff member. Any further clinical data, including the treatment provided, were written on diary cards and clinic forms and transcribed on to the Case Report Forms. If the physician found that the child had experienced an SAE, GSK Biologicals, MVI, and the Local Safety Monitor were promptly notified.

### Assessment of immunogenicity

Immunogenicity was assessed through the determination of blood parameters. Blood samples were taken by finger prick prior to the first dose (day 0), at month 3 and 14 for anti-HBsAg and anti-CS antibodies measurement.

Anti-HBsAg antibodies were tested at GSK Biologicals, Rixensart, Belgium, using an AUSAB EIA, Abbott, which had a cut-off of 10 mIU/ml. Antibodies specific for the circumsporozoite protein tandem repeat epitope were assessed by a standard, validated ELISA with plates adsorbed with the recombinant antigen R32LR that contains the sequence [NVDP(NANP)_15_]_2_LR. Briefly, R32LR protein was coated onto a 96-well polystyrene plate. Serum samples serial dilutions were added directly to the plate. The plates were washed and polyclonal rabbit anti-human IgG/HRP was added. After a final washing step and a color reaction with 3, 3',5,5' tetramethylbenzidine, the plates were read in an ELISA reader. The titers were calculated from a standard curve with the software SoftmaxPro (using a four parameters equation) and expressed as EU/ml. Anti-CS antibodies were tested at the CEVAC Laboratory, University of Ghent, Belgium. The cut-off for the anti-CS ELISA was 0.5 EU/ml.

### Statistical methods

#### Sample size calculation

Seventy-two evaluable subjects per group were required to demonstrate non-inferiority of RTS, S/AS02D compared to RTS, S/AS02A with 95% power, by comparing the upper limit of the 95% CI of the ratio of anti-CS GMTs (RTS, S/AS02A : RTS, S/AS02D) with a non-inferiority limit set at 3, assuming a log standard deviation of anti-CS GMTs of 0.72 (MAL-025; GSK data on file). Allowing for a chronic carriage rate of Hepatitis B virus of 15% and a drop out rate of 15%, it was necessary to enroll approximately 100 children per arm in this study.

#### Datasets used

Safety and reactogenicity were analyzed using the total cohort, defined as all enrolled subjects for whom safety and reactogenicity data were available. Immunogenicity was analyzed using the according to protocol (ATP) cohort, defined as all evaluable subjects (i.e. those meeting all eligibility criteria, complying with the procedures defined in the protocol, with no elimination criteria during the study) who tested negative for HBsAg at screening and for whom data concerning immunogenicity endpoint measures were available. These included subjects for whom assay results were available for antibodies against at least one study vaccine antigen component after Dose 3.

#### Analysis of immunogenicity

For each group, the seropositivity rate for anti-CS antibodies (proportion of subjects with anti-CS antibody titers of ≥ 0.5 EU/ml), and the seroprotection rate for anti-HBsAg antibodies (proportion of subjects with anti-HBsAg antibody titers of ≥ 10 mIU/ml) and their 95% Confidence Intervals (CI) were tabulated by vaccine dose at each time point (day 0 and months 3 and 14). Geometric mean titers (GMTs) for anti-CS antibodies (EU/ml), and anti-HBsAg antibodies (mIU/ml) with 95% CI were calculated for each group at each time point when a serology sample was taken.

The anti-CS vaccine response was calculated in two ways; the percentage of subjects with 3-fold increase in titers between day 0 and month 3 (day 90) and the geometric mean ratio (GMR, ratio of geometric means), which was the fold increase in antibody GMTs at day 90 compared to day 0.

In order to demonstrate non-inferiority for anti-CS and anti-HBsAg antibodies, the 95% CI for the ratio of GMTs (RTS, S/AS02A [0.25 ml dose] to RTS, S/AS02D [0.5 ml dose]) was computed using a one-way ANOVA model on the logarithm10 transformation of the titers. The 95% CI was also calculated using an analysis of covariance (ANCOVA) when adjusting for pre-vaccination titers. If the upper limit of the 95% confidence intervals of the ratio was below the clinical limits defining non-inferiority (three-fold difference), we can conclude that RTS, S/AS02D (0.5 ml dose) was non-inferior as compared to RTS, S/AS02A (0.25 ml dose).

#### Analysis of safety and reactogenicity

The percentage of subjects reporting solicited AEs within the seven-day follow-up period after vaccination was calculated with exact 95% CI by vaccine group, according to the type of AE (any and each specific solicited AE), their intensity (any and grade 3 only) and for general AE, according to their relationship to vaccination (any and those with suspected or probable relationship to vaccination). The percentage of subjects with any or related unsolicited symptom reported up to 30 days after vaccination, as tabulated per group. All serious adverse events occurring up to 30 days after dose three were listed for each treatment group.

## Results

**Figure 2:**
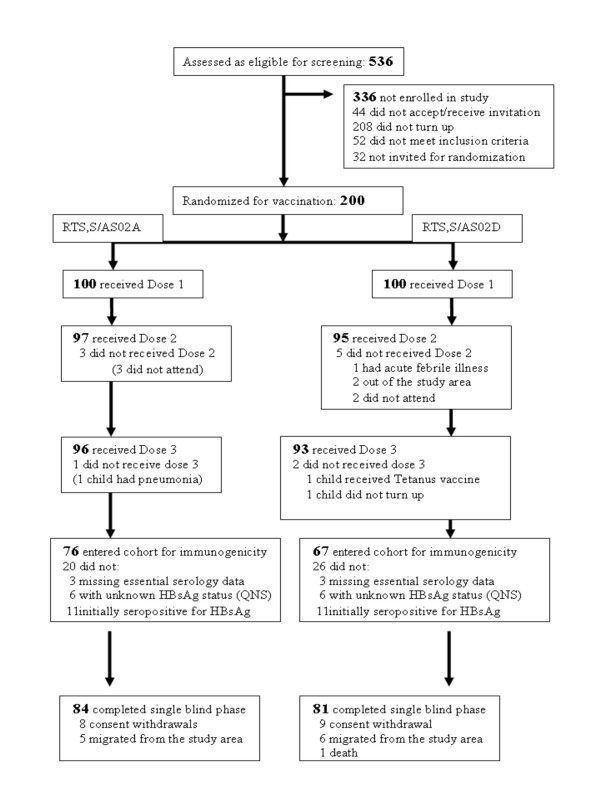
**Trial profile**. QNS: Quantity not sufficient.

A total of 200 children (100 per group), aged between 3 and 5 years were enrolled in this 14-month study from March 2004 to April 2005. The two groups were comparable with respect to mean age, sex and race (Table 1). Six subjects dropped out from the double-blind phase (day 0 to end of Month 3) and therefore, 194 subjects (97 per group) participated in the single-blind phase of the study (Month 4 to Month 14). Of the 194 subjects enrolled in the single-blind phase study, 165 subjects completed the study (81 in the RTS, S/AS02D group and 84 in the RTS, S/AS02A group). The reasons for withdrawal for the 29 subjects (16 in the RTS, S/AS02D group and 13 in the RTS, S/AS02A group) are presented in the (Figure 2).

**Table 1:** Summary of demographic characteristics (Total cohort)

**Characteristics**	**Parameters or Categories**	**RTS, S/AS02D N = 100**		**RTS, S/AS02A N = 100**		**Total N = 200**	
		
		**Value or n**	**%**	**Value or n**	**%**	**Value or n**	**%**
**Age (years) (at the start of the single-blind phase)**	Mean	4.2	-	4.2	-	4.2	-
	SD	0.99	-	0.89	-	0.94	-
	Median	4.0	-	4.0	-	4.0	-
	Minimum	3	-	3	-	3	-
	Maximum	6	-	6	-	6	-
	Unknown	3	-	3	-	6	-

**Gender**	Female	46	46.0	57	57.0	103	51.5
	Male	54	54.0	43	43.0	97	48.5

**Race**	Black	100	100.0	100	100.0	200	100.0

**Height (cm)**	Mean	99.5	-	99.9	-	99.7	-
	SD	7.26	-	7.26	-	7.24	-
	Median	99.5	-	101.0	-	100.0	-

**Weight (kg)**	Mean	15.4	-	15.8	-	15.6	-
	SD	2.44	-	2.51	-	2.48	-
	Median	15.4	-	15.8	-	15.6	-

**BMI (kg/m**^**2**^**)**	Mean	15.5	-	15.9	-	15.7	-
	SD	1.37	-	1.87	-	1.64	-
	Median	15.6	-	15.5	-	15.5	-

N: total number of subjects

n (%): number (percentage) of subjects in a given category

Value: value of the considered parameter

SD: standard deviation

BMI: Body Mass Index (kg/m^2^) = Weight/Height^2^

### Safety and reactogenicity

The incidence of symptoms, local and general solicited symptoms were not statistically different between the two groups (Table 2). The most frequently reported local symptom was pain, reported by 35% and 45% of subjects in the RTS, S/AS02D and RTS, S/AS02A groups respectively. The most frequently reported general symptom was irritability, reported by 28% and 25% of subjects in the two groups respectively. The incidence of symptoms in the seven days following vaccination did not increase with subsequent vaccinations in either group (data not shown). Grade 3 symptoms were reported with similar frequency in both groups.

**Table 2:** Overall incidence of solicited or unsolicited symptoms and incidence of individual solicited symptoms (any intensity and grade 3) in the 7-day follow-up period after vaccination (Total cohort)

**Group**		**RTS, S/AS02D (N = 100)**		**RTS, S/AS02A (N = 100)**	
		
		**%**	**95% CI**	**%**	**95% CI**
Any symptoms*		64.0	(53.8;73.4)	72.0	(62.1; 80.5)
	Grade 3	10.0	(4.9 ; 17.6)	13.0	(7.1; 21.2)

General symptoms*		43.0	(33.1; 53.3)	42.0	(32.2; 52.3)
	Grade 3	7.0	(2.9 ;13.9)	8.0	(3.5; 15.2)

Local symptoms*		39.0	(29.4;49.3)	55.0	(44.7; 65.0)
	Grade 3	3.0	(0.6; 8.5)	5.0	(1.6; 11.3)

Pain	Any	35.0	(25.7; 45.2)	45.0	(35.0; 55.3)
	Grade 3	2.0	(0.2; 7.0)	1.0	(0.0; 5.4)

Swelling	Any	9.0	(4.2; 16.4)	23.0	(15.2; 32.5)
	Grade 3	1.0	(0.0; 5.4)	4.0	(1.1; 9.9)

Drowsiness	Any	12.0	(6.4; 20.0)	8.0	(3.5; 15.2)
	Grade 3	4.0	(1.1; 9.9)	3.0	(0.6; 8.5)

Fever	Any	2.0	(0.2; 7.0)	1.0	(0.0; 5.4)
	Grade 3	0.0	(0.0; 3.6)	0.0	(0.0; 3.6)

Irritability	Any	28.0	(19.5; 37.9)	25.0	(16.9; 34.7)
	Grade 3	11.0	(5.6; 18.8)	10.0	(4.9; 17.6)

Loss of Appetite	Any	6.0	(2.2; 12.6)	3.0	(0.6; 8.5)
	Grade 3	1.0	(0.0; 5.4)	1.0	(0.0; 5.4)

N = number of subjects having received at least one dose and included in the analysis of reactogenicity

N (%) = number (percentage) of subjects presenting at least one type of symptom during the 7 day follow-up period after vaccination (day 0 to day 6)

*Any/General/Local symptom = includes all/general/local solicited and unsolicited symptoms occurring during the 7-day post vaccination follow-up period

The number of subjects with at least one unsolicited symptom reported during the 30-day follow-up and determined by the investigator to be related to vaccination was low (1.0% in the RTS, S/AS02D group vs 0% in the comparator group). We reported one SAE in the double-blind phase (RTS, S/AS02A group) and 10 (six in RTS, S/AS02D group and four in RTS, S/AS02A group) during the single-blind phase. None of the reported SAEs were considered to be causally related to vaccination. One volunteer that developed AIDS died during the follow-up period.

### Immunogenicity

One month following the last dose of vaccine (day 90), all subjects included in the ATP analysis of immunogenicity were seropositive with respect to anti-CS antibodies (Table 3). All subjects with paired pre- and post-vaccination samples showed a vaccine response with respect to anti-CS. GMTs were 191 EU/ml (95% CI 150–242) in recipients of RTS, S/AS02D compared to 180 EU/ml (95% CI 146–221) in recipients of RTS, S/AS02A. The RTS, S/AS02A:RTS, S/AS02D GMT ratio was 0.9 (95% CI 0.7–1.3). As the upper limit of the 95% CI of the GMT ratio between groups was below the pre-defined limit for non-inferiority of three (Table 3), non-inferiority of RTS, S/AS02D compared to RTS, S/AS02A was demonstrated in terms of the response to CS, and the primary objective was met. There was a decrease in anti-CS GMTs between month 4 and month 14 in both groups (191 vs 22 EU/ml and 180 vs 29 EU/ml in the RTS, S/AS02D and RTS, S/AS02A groups, respectively).

**Table 3:** Seropositivity rates and GMTs for anti-CS and Anti-HBs antibodies (ATP cohort for immunogenicity)

**Group**	**Timing**	**N**	**Seropositives**							**MIN**	**MAX**
					**95% CI**		**GMT**	**95% CI**			
			**n**	**%**	**LL**	**UL**		**LL**	**UL**		
**GMTs anti-CS antibodies (EU/mL)**											

RTS, S/AS02D	Pre	67	9	13,4	6,3	24	0,3	0,3	0,4	< 0,5	8
	PIII(M3)	67	67	100	94,6	100	191	150	242	14	1314
	PIII(M14)	60	60	100	94	100	22	15	32	0,5	543

RTS, S/AS02A	Pre	76	10	13,2	6,5	22,9	0,3	0,3	0,4	< 0,5	8
	PIII(M3)	76	76	100	95,3	100	180	146	221	16	1277
	PIII(M14)	67	67	100	94,6	100	29	22	39	1	323

**GMTs anti-HBs antibodies (mIU/mL)**											

RTS, S/AS02D	Pre	67	6	9,0	3,4	18,5	8	5	11	< 10	2211
	PIII(M3)	67	67	100	94,6	100	23978	17896	32127	985	446880
	PIII(M14)	60	60	100	94,0	100	5184	4043	6646	653	100110

RTS, S/AS02A	Pre	76	5	6,6	2,2	14,7	6	5	8	< 10	1759
	PIII(M3)	76	76	100	95,3	100	17410	13322	22752	1265	823500
	PIII(M14)	66	66	100	94,6	100	5024	3918	6443	877	260700

N: number of subjects with available results; GMT: geometric mean antibody titer calculated on all subjects; MIN/MAX: Minimum/Maximum; n (%): number (percentage) of subjects with titer ≥ 0.5 EU/mL for Anti-CS or with titer ≥ 10 mIU/mL for Anti-HBsAg; 95% CI: 95% confidence interval; LL: Lower Limit, UL: Upper Limit; Pre: Pre-vaccination; PIII(M3): blood sampling taken 3 months after the first dose of vaccine; PIII(M14): blood sampling taken 14 months after the first dose of vaccine

With respect to anti-HBsAg, all subjects were seroprotected at day 90 (Table 3), and the GMTs were 23978 mIU/ml (95% CI 17896 – 32127) in RTS, S/AS02D recipients and 17410 mIU/ml (95% CI 13322–22752) in RTS, S/AS02A recipients. The RTS, S/AS02A:RTS, S/AS02D GMT ratio was 0.7 (95% CI 0.5–1.1). As the clinical limit defining non-inferiority was defined as an upper limit of the 95% CI of 3, non-inferiority was concluded.

## Discussion

In most areas of stable malaria transmission in Africa, the prevalence of infection increases over the first few years of life and classically peaks around 5 years of age. This has led to the long term notion that immunity against the parasite tends to develop slowly, and that the main burden of malaria is in children around that age. However, emerging evidence shows that, a very significant proportion of deaths and severe episodes due to *P. falciparum *malaria concentrate in children less than 18 months of age [10,12]. Any control tool that aims towards having a significant public health impact should therefore be delivered as early as possible in life so as to prevent the significant morbidity and mortality associated with *P. falciparum *infection during the first 18 months.

Many African countries where malaria is endemic struggle with limited human and financial resources to run efficient National Health Services (NHS). The Ministry of Health of Mozambique estimates that approximately 50% of its population has no access to the services of its NHS. This rather common fact reminds us of the limitations of treatment of malaria cases and emphasizes the need to deploy preventive tools. One relatively well-functioning health service operating in developing countries is the WHO EPI, which manages to deliver a number of different vaccines, even to remote populations, during the first year of life. Renewed efforts to strengthen this system through GAVI/The Vaccine Fund, are yielding significant results with respect to vaccine delivery and the attainment of high coverage rates. Taking into consideration these elements, the Clinical Development Plan (CDP) of RTS, S malaria vaccine for public health use in Africa, aims to deliver vaccines as early as possible in life to prevent mortality due to malaria during the first months of life. Furthermore, the CDP ideally aims to deliver a malaria vaccine alongside other vaccines within the EPI schedule, thereby making use of already existing contacts between the health services and the target population.

RTS, S/AS02D, a new formulation of the RTS, S candidate malaria vaccine, has been produced in order to match the volume of all other vaccines administered during the first year of life and therefore facilitate its administration and inclusion into the EPI schedule.

This study shows that the safety and reactogenicity profile observed with RTS, S/AS02D is similar to that of RTS, S/AS02A, as well as that previously documented in other studies for RTS, S/AS02A [5]. Although there are no established immune correlates of protection against malaria sporozoites and liver stage parasite, it is widely believed, on the basis of preclinical studies in animal models and clinical investigators, that both humoral and cell-mediated immune responses are involved in protective immunity [13,14]. In this trial conducted in African children we were not able to adequately measure T-cell immunity. Humoral responses were therefore used as the best available and relevant tool for this clinical bridge between the two formulations of RTS, S. Using this analysis, the study shows that the immunogenicity of the two formulations are similar, for both anti-CS and anti-HBsAg humoral responses. This result allows us to proceed with the CDP, caring studies in infancies in parallel with the EPI immunization. Some of these studies are now ongoing.

## Conclusion

In conclusion, our data show that the new formulation, RTS, S/AS02D (0.5ml) is as safe and immunogenic as the existing formulation, RTS, S/AS02A (0.25 ml), in this Mozambican pediatric population. These results allow us to proceed further in the clinical development of this candidate malaria vaccine and initiate Phase I/IIb trials in the infant population.

## Conflict of interest statement

MVI supports the development and testing of several malaria vaccines that can be seen as competitors. AL, ML, CPC, MK, M-CD, WRB, and JC are employees of GSK Biologicals. AO was on job training at GSK. AL, WRB, and JC own shares in GSK. JC and WRB are listed as the inventors of patented malaria vaccines; however, neither individual holds a patent for a malaria vaccine. None of the other authors in this paper have declared conflicts of interest.

## Authors' contributions

EVM, JS, MN, and PLA were involved in all phases of the study and were primarily responsible for the implementation. AL led the clinical team at GSK Biologicals. JA and ML, led the data analysis. EVM and JS were responsible for field, hospital activities and safety surveillance. JM, MN, MS and CG were the program managers. IM and YL coordinated all laboratory work at CISM. AO, was a clinical monitor at GSK Biologicals, MK was responsible for coordinating all laboratory work at GSK, M-CD, was the malaria vaccine project manager at GSK Biologicals. CPC was the scientific writer at GSK Biologicals, RT and FD, contributed to the design of the study, and interpretation. WRB, is the Vice President – Clinical Development Emerging Diseases Vaccines, at GSK Biologicals, JC, is Vice President Emerging Diseases and HIV Vaccines and heads the malaria vaccine Research and Development at GSK Biologicals. PA is the CISM scientific director. The manuscript was written by EVM and PA, with input from all other authors.
